# Tango-therapy vs physical exercise in older people with dementia; a randomized controlled trial

**DOI:** 10.1186/s12877-023-04342-x

**Published:** 2023-10-24

**Authors:** Lucía Bracco, Arrate Pinto-Carral, Linda Hillaert, France Mourey

**Affiliations:** 1 Inserm U1093-Cognition, Action and Sensorimotor Plasticity, Faculty of Sport Sciences, University of Burgundy, 21078 Dijon, France; 2 SALBIS Research Group, Faculty of Health Sciences, Nursing and Physiotherapy Department, Universidad de León, 24401 Ponferrada, Spain; 3 Centre Hospitalier Gériatrique du Mont d’Or, Albigny Sur Saône, France

**Keywords:** Abilities of daily living, Dance-therapy, Dementia, Gait speed, Older adults, Quality of life

## Abstract

**Background:**

Dementia is a growing health concern that affects millions of people worldwide. Gait and mobility disorders are often present and represent a major risk factor for falls. The purpose of this study was to investigate the effectiveness of tango-therapy in gait speed, functional mobility, balance, falls, ability to perform activities of daily living and quality of life.

**Methods:**

A randomised controlled trial with 31 participants living in a specialised dementia unit, aged 65 to 93 years old, who were randomly assigned to tango group (IG) or physical exercise group (CG). The primary outcome was gait speed and Timed Up and Go test. The secondary outcomes include the Short Physical Performance Battery, the ability to perform activities of daily living (Katz Index) and quality of life (Quality of life in Alzheimer Disease). Measurements were performed at baseline, and after one and three months of training.

**Results:**

After 3 months, IG improved gait speed (*p* = 0.016), implying a statistically significant difference between groups in favour of IG (*p* = 0.003). CG significantly worsened the time to complete the TUG (*p* = 0.039). Both groups declined in their ability to perform activities of daily living, being statistically significant only in the CG (*p* < 0.001).

**Conclusion:**

Tango interventions showed efficacy in improving gait speed and in mitigating the decline in functional mobility and ADL skill capacities. Allowing older people with dementia access to non-pharmacological interventions may be a successful strategy to prevent functional decline.

**Trial registration:**

Registered at ClinicalTrials.gov (ID: NCT05744011).

## Introduction

As the global population ages, dementia is becoming an increasingly prevalent health issue that affects millions of people worldwide, with devastating consequences for both the individuals living with the condition and their families, as well as profound social, economic, and health implications for society as a whole [1]. One of the most characteristic signs of dementia is a decline in cognitive function, including memory loss, difficulty with language, impaired judgment, behavioural issues, and problems with activities of daily living (ADL). These aspects have an impact on physical performance, triggering balance and gait disorders [2], which worsen the overall condition and represent a major risk factor for falls [3].

Dementia is one of the main reasons for institutionalization, and over 50% of residents of nursing homes present cognitive impairment. Admission to a facility is substantially associated with functional decline, which is further amplified in the presence of dementia syndrome [4]. Indeed, the loss of the stimulus of daily life and of spatiotemporal reference points results in a decrease in activity, which in the short term causes a loss of capacities due to disuse [5]. In order to maintain each person's level of autonomy, it is crucial to study new approaches for preventing physical and cognitive decline. Non-pharmacological interventions are multifaceted actions aimed at preserving and even enhancing the cognitive, physical, psychological, and social abilities and, more broadly, the quality of life (QoL) of patients [6].

Music and dance have been proven to be useful non-pharmacological interventions in several contexts, through the training of attention, memory, hearing, rhythm, coordination, balance, and self-perception of the body in space [7]. These activities also provide the opportunity to explore feelings, forge relationships, and enhance self-esteem. Dance is particularly valued because it integrates emotions, social connection, and sensory stimulation in a physical and creative activity, resulting in rich environmental conditions [8].

Tango holds a unique place among all dances because of the cultural and emotional resonance it carries as well as the way it mobilizes the motor cognitive functions [9]. Tango is a couple's social dance that emphasizes strong corporal expression through an emotional attitude and connection with the other person through a closed embrace [10]. As the partners synchronize, the steps naturally follow one another in all directions. The basis of this style is the normal gait: each step is preceded by a subtle anticipatory movement which is communicated to the partner through the connection in the embrace. The rhythm, pacing, and direction change to naturally follow the music. The regular practice of this dance brings positive effects on several aspects of health [9].

Few studies have analysed the effects of dance on older people with dementia, in terms of gait and functional mobility [11]. Despite evidence from empirical data and from field observations, current scientific knowledge does not establish an effect with a sufficient degree of confidence [12]. A previously multicentre quasi-experimental study on older people living in nursing homes with and without dementia reported feasibility and suggested effect of tango therapy interventions on QoL [13]. The purpose of this study was to investigate the effectiveness of tango-therapy in gait speed, functional mobility, balance, falls, ADL and QoL. The hypothesis posited that tango-therapy, as a multicomponent intervention, would prove more effective than a physical exercise program—encompassing strength training, balance exercises, and stretching—, in older individuals with dementia.

Methods.

### Study design

This was a 3-month parallel randomized controlled trial study with assessment at baseline, after one month and at the end of the 3-month intervention period. The study was conducted between December 2022 and March 2023, in the Protected Life Units (PLUs), which belong to the Pavillon Jacques Chauvire nursing home, belonging to the Mont d'Or Geriatric Hospital, France. PLUs are small units that provide accommodation for older people with dementia who, for the most part, present neuropsychiatric symptoms that make it impossible to house them in conventional units.

### Ethics

The protocol was designed following the recommendations of the Consolidated Standards of Reporting Trials statements [14]. The study was conducted following the Declaration of Helsinki, was approved by the Research Ethics Committee of the University of Burgundy (CERUBFC-2022–09-29–031) and was registered at ClinicalTrials.gov (ID: NCT05744011). Each person likely to take part in this research was informed and a summary document on the main characteristics of the study was given during the inclusion visit. The written informed consent statement was given for immediate signature or after a reflection period. In case of doubt about the patient's ability to give consent, caregivers and the healthcare team were asked to help measure the expression of the person's autonomy and presumed wishes. In the case of persons under legal protection, the magistrate and the competent judge were informed, and authorization was requested.

### Participants

The principal investigator recruited the participants among the residents of the PLUs. The inclusion criteria were being aged 65 or over, a permanent resident in a PLU, able to walk 10 m without human assistance and having given consent to participate in this study. The exclusion criteria were the presence of a medical contraindication, being bedridden or patients in terminal care. The participants were randomised by the principal investigator in the intervention group (IG) or in the control group (CG) using the option “random sample of cases” of IBM SPSS statistics software (Version 25).

### Intervention

People assigned to the IG completed a therapeutic tango program, implemented by ABB Reportages (to know more: https://youtu.be/XJA2f89MneU), consisting of two weekly 60-min sessions for three months. The participants had no prior experience with the therapeutic tango program. Every session was conducted in groups, ensuring no group exceeded 15 participants. Twice a week, all participants gathered in a single room for the intervention, fostering an atmosphere of camaraderie and group learning. The sessions were led by nursing staff who previously received training in therapeutic tango by the University of Burgundy (to know more: https://youtu.be/CNxyr1Wv1bs). Nursing staff was composed of nurses, nursing assistants, psychologists, physical activity teachers, and psychomotor therapists. A list of reproductions of tango, waltzes and milonga was available, to adapt the music to the different types of exercise. Twice a month the sessions were conducted by a team of dance-movement-therapist and musician, in order to maintain motivation, bring novelty and give cohesion to the interventions.

The main objective of the interventions was to create a favourable environment that facilitates the movement of people. For this, the facilitator worked from non-verbal communication, relying on the remaining capacities of each participant. The second objective was to work on walking and functional mobility, through tango steps. The therapeutic tango session was composed basically as follows:

1. Scenario and warm-up: seated exercises to mobilise the lower and upper limbs, the head and the trunk as well as singing to warm up the voice and promote social ties.
2. Central part: dance standing or sitting. Solo, couple or group. Different aspects of the tango were practiced: forward, backward, sidestep, square, rectangle, *ochos*, etc. Changes in pace, speed and direction. Improvisation through spontaneous expression. Physical connection through hugging or other interactions is also an important aspect of the intervention.
3. Cooling down: seated rituals such as chanting and breathing exercises.
4. The farewells: moment of exchange and feedback between the participants and the staff.

### Control group

People assigned to the CG completed a physical exercise program. The choice of this activity was made considering the extensive body of research that supports the assumption that people with Alzheimer's disease or other dementias can benefit significantly from physical exercise [15]. The program consisted in two weekly 60-min sessions for three months. The sessions were led by a physical exercise teacher, experienced in working with older people. The intervention was conducted in a group setting, and the group could not exceed 15 people. At the beginning of the session, participants were arranged in a large circle. Strength, balance, and stretching activities were conducted individually, in pairs, or in groups. Music was not allowed during the sessions, which were composed as follows:

1. Warm-up: the session begins with seated and standing exercises to engage all major joints, enhancing blood circulation and joint flexibility. Examples of warm-up exercises: seated shoulder circles, ankle pumps, arm raises.
2. Central part: this segment includes dynamic exercises using scarves or balls to boost coordination and cognitive stimulation. Examples of central part exercises: scarf toss, ball squeezes, chair squats, balloon volley.
3. Return to calm: the session concludes with gentle stretches and self-massage to release muscle tension and promote relaxation. Examples of cool-down exercises: neck stretches, arm and shoulder stretch, gentle leg stretch, deep breathing.

The exercises are designed for safety, engagement, and enjoyment, considering the needs of older people with dementia. The session aims to enhance both physical and cognitive well-being.

### Outcome measures

Medical history and sociodemographic data were collected. The Mini Mental State Examination (MMSE) was used to assess cognitive performance [16]. Considering the pronounced cognitive impairment among residents in PLUs, and the frequent occurrence of a floor effect when assessing their cognitive abilities with the MMSE, we classify the stage of dementia as “severe” for scores ranging from 0 to 7, and as “moderate” for scores range from 8 to 20 on the MMSE [17]. The Neuropsychiatric Inventory (NPI) was used to assess dementia-related behavioural symptoms [18], and the Charlson Index of Comorbidity (CCI) to assess overall comorbidity [19]. History for falls in the previous three months and during the 3-month intervention was collected.

The study involved collecting outcome measurements at three different time points: before the beginning of the intervention, one month later, and at the conclusion of the intervention. The principal investigator provided guidance and support to the healthcare staff responsible for data collection, all of whom were well-acquainted with each participant and familiar with their daily routines. For this reason, it was not possible to blind the assessment.

### Primary outcomes

The 4-m gait speed is a physical performance test used to assess an individual's mobility and functional ability, particularly in older adults. During the test, the person is asked to walk 4 m at their usual pace, with or without an assistive device like a cane or walker, while being timed with a stopwatch. The test is performed twice, and the best time is recorded. The test is quick, easy and has been found to be a good predictor of various health outcomes, such as disability, hospitalisation, and mortality risk, as well as overall physical performance and frailty in older adults. Its reliability was evaluated in older people with cognitive impairment, showing an intraclass correlation coefficient (ICC) for inter-rater reliability that reached values of 0.96 and 0.91 for the test–retest study [20].

Timed Up and Go test (TUG-test) was used to assess functional mobility. The test involves timing how long it takes for a person to get up from a chair, walk three meters, turn around, walk back to the chair, and sit down again. The test procedure was as follows: the participant sat in a chair with their back against the back the chair. It was explained to them that they were to get up when instructed, walk at their pace to the mark on the ground three meters away, turn around, and walk back to the chair to sit down. The evaluator started timing when they instructed the patient to get up from the chair and stopped when the patient returned to the starting position. Two attempts were allowed, and the best time was recorded. The relative reliability of TUG-test has been tested in older people with dementia showing an ICC > 0.90 [21].

### Secondary outcomes

Intervention attendance was noted and computed as follows: ([number of sessions attended/total number of sessions] × 100).

Following each session, the intervention facilitator asked the participants about their subjective feeling of well-being using the EVIBE (Échelle d’évaluation instantanée du bien-être, Scale of instantaneous well-being), which is a visual analogue scale. They were asked to rate their current feeling of well-being on a graduated ruler from 1 to 5 in response to the question "How do you feel now?". A rating of 5 indicated the strongest feeling of well-being, while a rating of 1 indicated the weakest. This scale has been validated in older adults with severe cognitive impairment and has demonstrated a high level of intrajudge reliability (ICC between 0.79 and 0.90; *p*< 0.001) [22].

The Short Physical Performance Battery (SPPB) is a test that assesses physical function in older adults. It is commonly used in clinical settings and research studies to evaluate an individual's lower extremity function, balance, and gait speed. The SPPB consists of three tests. Balance tests: the participant is asked to maintain balance in three different feet positions—side-by-side, semi-tandem, and tandem—for up to 10 s each. Gait speed test: The participant is timed while walking four meters at their usual pace. Chair stand test: The participant is timed while standing up from a chair and sitting down again five times as quickly as possible. Each test is scored on a scale of 0–4. The scores from each test are added together to give an overall SPPB score, which ranges from 0 (poor performance) to 12 (excellent performance). The SPPB has been shown to be a reliable and valid tool for assessing physical function in older adults and can help healthcare professionals identify individuals at risk of mobility disability and functional decline [23].

The Katz Index of Independence in ADL is a tool used to evaluate an individual's ability to perform basic self-care tasks in six basic domains: bathing (the ability to wash oneself in a tub or shower), dressing (the ability to put on and take off clothing, including shoes and socks), toileting (the ability to get on and off the toilet and clean oneself), transferring (the ability to move from a bed to a chair or wheelchair and vice versa), continence (the ability to control bladder and bowel movements), feeding (the ability to feed oneself, including using utensils and preparing food). Each of these activities was rated by the nursing staff on a three-point scale (dependent, partially dependent, and independent) based on the amount of assistance required to perform the task. The scores are then added up to provide an overall measure of functional status, with a maximum score of 6 indicating complete independence in all six ADLs. The Katz Index is a simple and effective tool for assessing functional status and can be used to guide care planning and monitor changes in an individual's ability to perform basic self-care tasks over time [24].

The Quality of Life in Alzheimer's Disease (QoL-AD) is a questionnaire designed to assess the QoL of people with Alzheimer's disease and related dementias, consisting of 13 items organised into physical health, mood, living situation, memory, family, friends, and ability to carry out daily tasks. Response options range from 1 (poor), 2 (fair), 3 (good) to 4 (excellent), for a total score of 13–52. Higher scores indicate better QoL. The questionnaire was administered by the principal investigator or the nursing staff responsible for data collection. Both the participant and their professional caregiver provided input by completing separate questionnaires, which were then combined into a composite score as follows (2 × participant score + 1 × caregiver score)/3, giving greater weight to the participant's responses. The QoL-AD questionnaire was assessed in French for its validity and reliability [25]. The results showed good internal consistency, with a Cronbach's alpha coefficient of 0.70 or higher, and good reliability, with an ICC of over 0.80 for patient and caregiver questionnaires administered at a 2-week interval [26]. Test–retest reliability was also good, with a Cronbach's alpha coefficient of 0.8930 for older individuals with severe cognitive impairment. Furthermore, there was a significant correlation between QoL-AD and the Activities of Daily Living Inventory (ACDS-ADL) (*p* < 0.001) and Health Status Questionnaire (HSQ-role-physical) (*p*< 0.01), indicating good construct validity. The QoL-AD is a valid and reliable tool for people with an MMSE score greater than 2 [27].

### Data analysis

Descriptive statistics were employed to summarize both qualitative and quantitative variables. Categorical variables were expressed as percentages (%), while means, standard deviations, and their 95% confidence intervals were computed for quantitative variables. Comparisons of categorical variables were conducted using the chi-square test.

The normality of continuous data distribution was assessed through the Shapiro–Wilk test. For non-parametric analyses, the Mann–Whitney U test was utilized for independent samples, and the Friedman test was applied for repeated measures. Post hoc comparisons were performed using the Wilcoxon signed-rank test.

Parametric analyses involved Repeated Measures ANOVA. In-depth intragroup and intergroup comparisons were performed through a 2-way ANOVA. This analysis encompassed two groups and three measurement moments, considering repeated measurements over time as a within-subject factor and groups as a between-group factor. The effect size for F-tests was gauged by the partial Eta squared coefficient (ηp2), where values of 0.01, 0.06, and 0.14 indicated small, medium, and large effect sizes respectively. Post-hoc comparisons were adjusted with Bonferroni's correction and adjusted p-values. Effect sizes were determined using Cohen's d, categorized as very small (d < 0.20), small (0.20 ≤ d < 0.50), medium (0.50 ≤ d < 0.80), or large (d ≥ 0.80). The alpha level was set at *p* < 0.05 within a 95% confidence interval (CI), and statistical analyses were performed using IBM SPSS version 25.

## Results

**Fig. 1:**
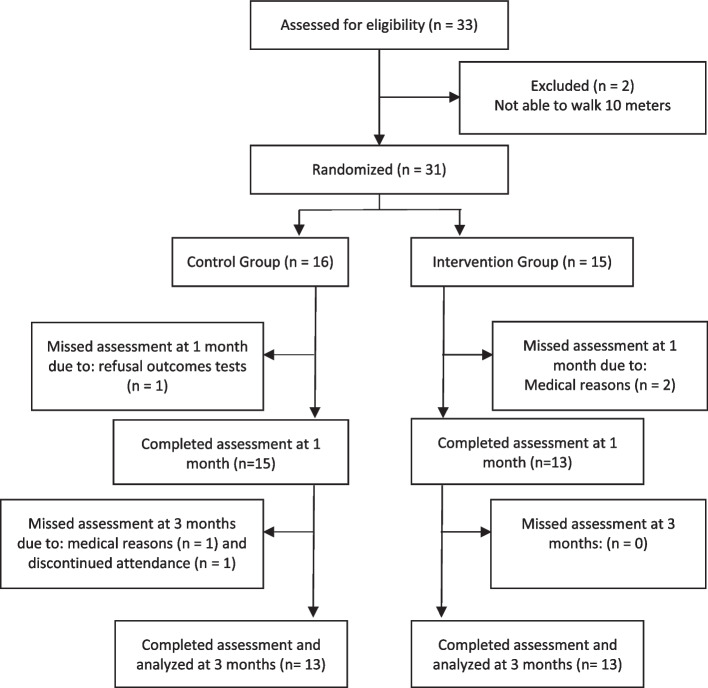
Flowchart for enrolment, allocation and follow-up of participants

Figure 1 shows the flow of the participants through the study. A total of 31 residents met the inclusion criteria. The study concluded with 26 participants successfully completing the protocol, undergoing assessment, and being included in the analysis of outcomes.

### Sample characteristics

Table 1 presents the primary characteristics of the baseline sample and each group. The average age of the participants was 83 ± 6.6 years, and 84% of them were female. The average score on the MMSE was 5.3 ± 5.3, and on the Charlson Index was 7.5 ± 1.7* (*only variable that showed statistically significant differences between the groups (*p* = 0.033)*).*

**Table 1:** Baseline characteristics of participants assigned to each study group

Characteristic		Total Simple (*n* = 31)	IG (*n* = 15)	CG (*n* = 16)	*p*
**Age, mean ± SD**		83 ± 6.6 (65–93)	81 ± 8 (65–93)	85 ± 5 (78–93)	0,201
**Sex, females, n (%)**		26 (84)	11 (73)	15 (94)	0.122
**MMSE, mean ± SD**		5.3 ± 5.3 (0–19)	7 ± 5 (0–19)	4 ± 5 (12–0)	0.148
**Stage of dementia n (%)**	**Severe**	18 (58)	7 (47)	11 (69)	0.189
	**Moderate**	13 (42)	8 (53)	5 (31)	
**Charlson Index, mean ± SD**		7.5 ± 1.7 (3–11)	7 ± 2 (3–9)	8 ± 1 (6–11)	0.033*
**NPI**		33 ± 20	31 ± 24	36 ± 17	0.243

*IG* Intervention group, *CG* Control group, *SD* Standard deviation, *MMSE* Mini mental state examination; *p < 0.05 based on independent samples Mann Whitney U-test for continuous variables or on Chi squared test for categorical variables

### Intervention attendance and well-being

It is worth noting that the program experienced a 20-day interruption following the first post-test due to a Covid-19 outbreak. This had an impact on the frequency and number of activities, which may have influenced the results of both groups. However, it is important to acknowledge that both groups received an equal number of sessions, at the same frequency, on the same days, and at the same time. Throughout the study, both groups took part in a total of 21 sessions—8 during the first month and 13 during the final two months of the intervention. Nonetheless, on average, each participant attended 19 ± 1.5 sessions, resulting in an average attendance rate of 90%. Following every session, the participants' self-reported sense of well-being was evaluated using EVIBE, and the mean score obtained was 3.9 ± 0.5 on a five-point scale. The attendance rates of both groups did not show any significant differences (IG = 19.46 ± 1.2; CG = 18.92 ± 1.71; *p* = 0.361), and there was also no significant difference between the two groups in terms of subjective well-being scores after each session (IG = 3.84 ± 0.48; CG = 3.9 ± 0.53; *p* = 0.755).

### Intervention effects

**Fig. 2:**
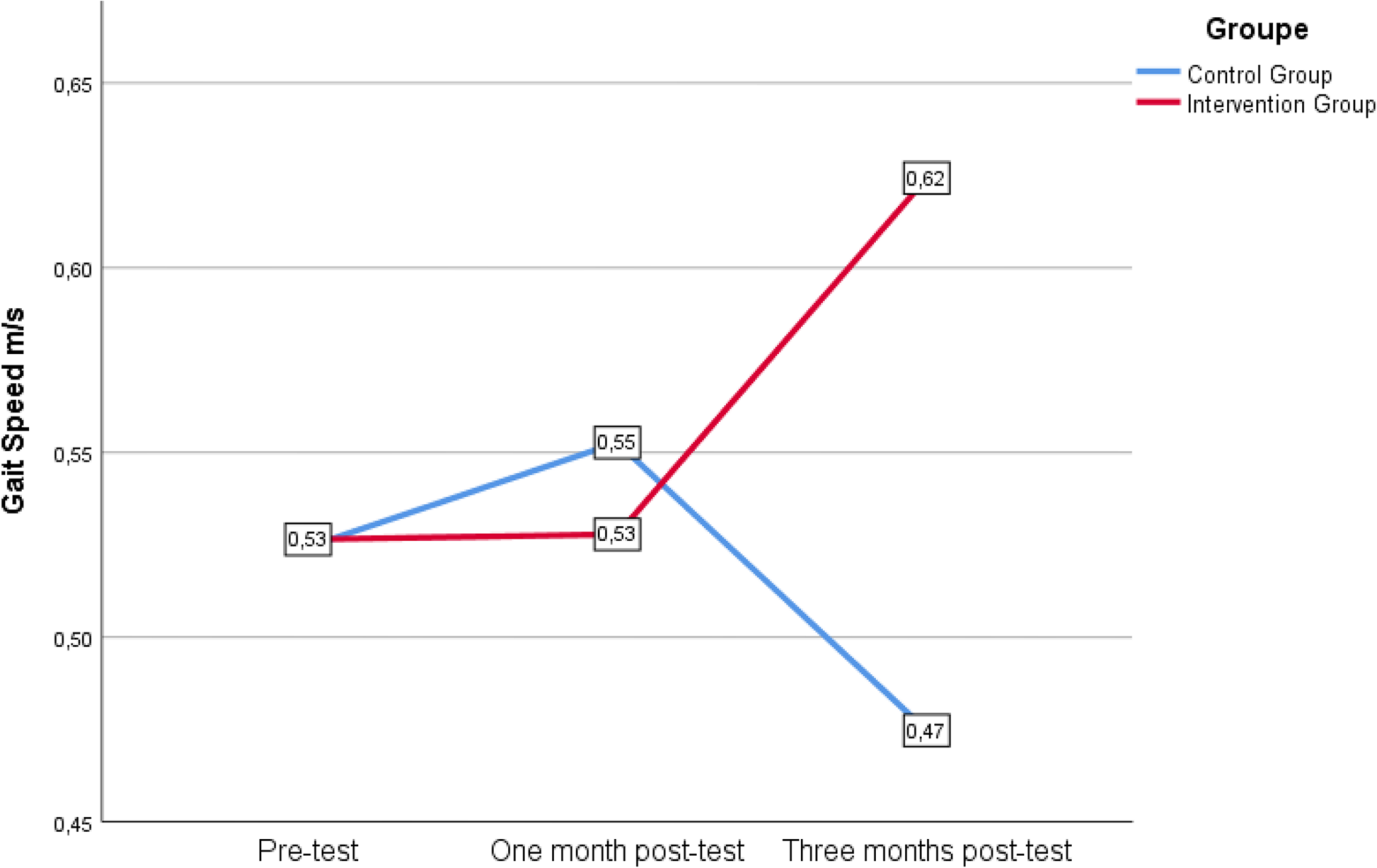
Evolution of gait speed for participants who completed 3 assessments (*n* = 26)

Table 2 shows the primary outcomes at baseline, one month, and three months post-test, while Table 3 illustrates the intragroup and intergroup differences in outcomes. The IG showed a significant improvement in gait speed, with a large overall effect size, achieving an increase of nearly 0.1 m/s (F_(2)_ = 4.939, *p* = 0.016, ηp^2^ = 0.292, β-1 = 0.756). Additionally, the repeated measures ANOVA demonstrated a between-groups statistically significant differences with a large overall effect size (F_(2)_ = 6.758, *p* = 0.003, ηp^2^ = 0.220, β-1 = 0.901). According to the Bonferroni correction, this difference was specifically observed in the three-month post-test, with a mean difference of 0.149 m/s (*p* = 0.049, d = 0.85). Figure 2 illustrates the evolution of gait speed according to the group.

**Table 2:** Principal outcomes at baseline, at one month and at three months post-test

Variables		IG			CG		
		T1	T2	T3	T1	T2	T3
**Gait speed (m/s), mean ± SD**		0.53 ± 0.21	0.53 ± 0.21	0.62 ± 0.22	0.52 ± 0.16	0.55 ± 0.18	0.47 ± 0.14
**Katz Index, mean ± SD (0–6)**		2.9 ± 1.3	2.5 ± 1.1	2.27 ± 0.88	2.7 ± 1.2	2.7 ± 1.1	2 ± 0.91
**QoL-AD, mean ± SD**	Participant (13–52)	36.9 ± 3.9	38.7 ± 4.8	37.8 ± 5.3	34.6 ± 4	39.5 ± 3.6	36.9 ± 5.4
	Caregiver (13–52)	30.7 ± 7.5	34.3 ± 6.5	34.5 ± 4.6	31.2 ± 5.2	33.5 ± 6.6	35.2 ± 7
	Composite (13–52)	35 ± 2.6	37.4 ± 3.4	36.7 ± 3.9	33.2 ± 3.5	37.5 ± 3.7	36.9 ± 3.9
**SPPB, mean ± SD**	Static Balance (0- 4)	1.5 ± 1.1	1.5 ± 1.1	1.23 ± 1	1.4 ± 1.3	1.7 ± 0.9	1.54 ± 1.2
	Gait speed (0–4)	2 ± 1	1.9 ± 1	2.4 ± 1.1	2 ± 0.9	2.1 ± 1	1.6 ± 0.7
	Sit to stand (0–4)	0.5 ± 0.5	0.7 ± 0.6	0.5 ± 0.5	0.9 ± 1	1.1 ± 1	1 ± 0.9
	Total (0–12)	4 ± 2	4.3 ± 2.4	4.3 ± 2.2	4.3 ± 2.1	4.5 ± 2.2	4.1 ± 1.8
**TUG (s), mean ± SD**		27.16 ± 14	23.56 ± 10.73	24.75 ± 6.04	26.69 ± 10.72	25.6 ± 13.9	29.36 ± 11.92

*IG* Intervention group, *CG* Control group, *SD* Standard deviation, *SPPB* Short Physical Performance Battery, *m/s* Meters par seconds, *TUG* Timed Up and Go test, *QoL-AD* Quality of Life in Alzheimer Disease, *T1* Pre-test, *T2* 1-month post-test, *T3* 3-month post-test

**Table 3:** Outcome differences intragroup and between groups

Variables		Intergroup comparison (*p*)			Intragroup comparison (*p*)	
					TANGO	APA
**Gait speed (m/s), mean ± SD**		0.003*^c^			0.016*^c^	0.119^c^
**Katz Index, mean ± SD (0–6)**		0.406^c^			0.150^c^	< 0.001*^c^
**QoL-AD, mean ± SD**	Participant (13–52)	0.528^c^			0.391^c^	0.260^c^
	Caregiver (13–52)	0.855^c^			0.121^c^	0.184^c^
	Composite (13–52)	0.859^c^			0.074^c^	0.416^c^
		T1/T1	T2/T2	T3/T3		
**SPPB, mean ± SD**	Static Balance (0- 4)	0.960^a^	0.569^a^	0.579^a^	0.401^b^	0.902^b^
	Gait speed (0–4)	0.960^a^	0.687^a^	0.091^a^	0.045*^b^	0.115^b^
	Sit to stand (0–4)	0.390^a^	0.459^a^	0.243^a^	0.368^b^	0.727^b^
	Total (0–12)	0.724^a^	0.614^a^	0.960^a^	0.513^b^	0.794^b^
**TUG (s), mean ± SD**		1^a^	0.843^a^	0.246^a^	0.205^b^	0.039*^b^

*IG* Intervention group, *CG* Control group, *SD* Standard deviation, *SPPB* Short Physical Performance Battery, *m/s* Meters par seconds, *TUG* Timed Up and Go test, *QoL-AD* Quality of Life in Alzheimer Disease, *T1* Pre-test, *T2* 1-month post-test, *T3* 3-month post-test. *p < 0.05 based on, ^a^Mann–Whitney U test for non-parametric independent samples, ^b^Friedman test for non-parametric repeated measures, and ^c^Repeated Measures ANOVA for parametric data

**Fig. 3:**
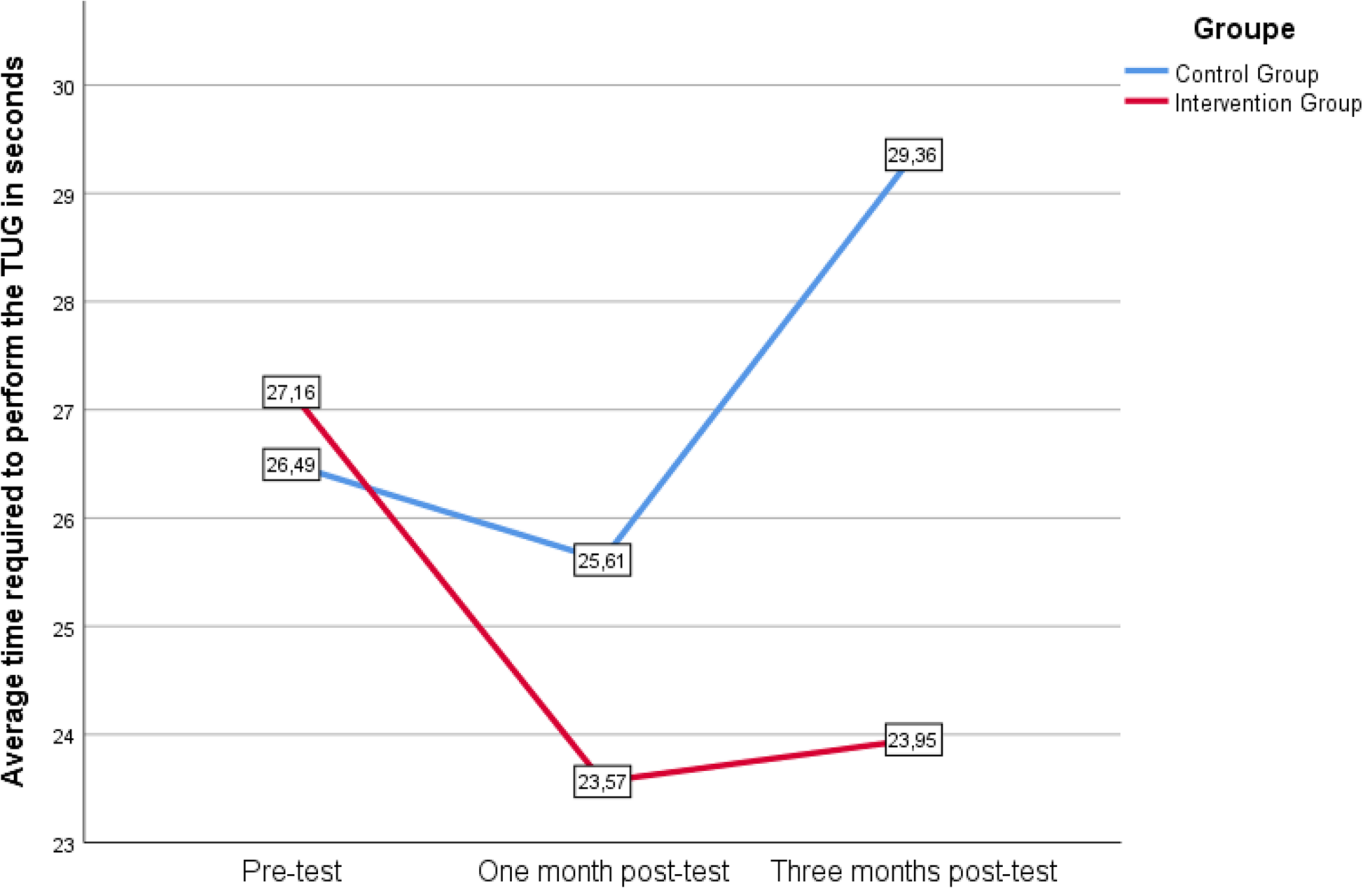
Evolution of the time required to perform the TUG-test for participants who completed 3 assessments (*n* = 26)

Regarding functional mobility, the Friedman test indicated that the CG significantly worsened its time to complete the TUG test (χ2_(2)_ = 6.5, *p* = 0.039). However, post-hoc comparisons using the Wilcoxon test did not reveal significant differences between the various measurements. Figure 3 illustrates the TUG performance trajectory for both groups.

**Fig. 4:**
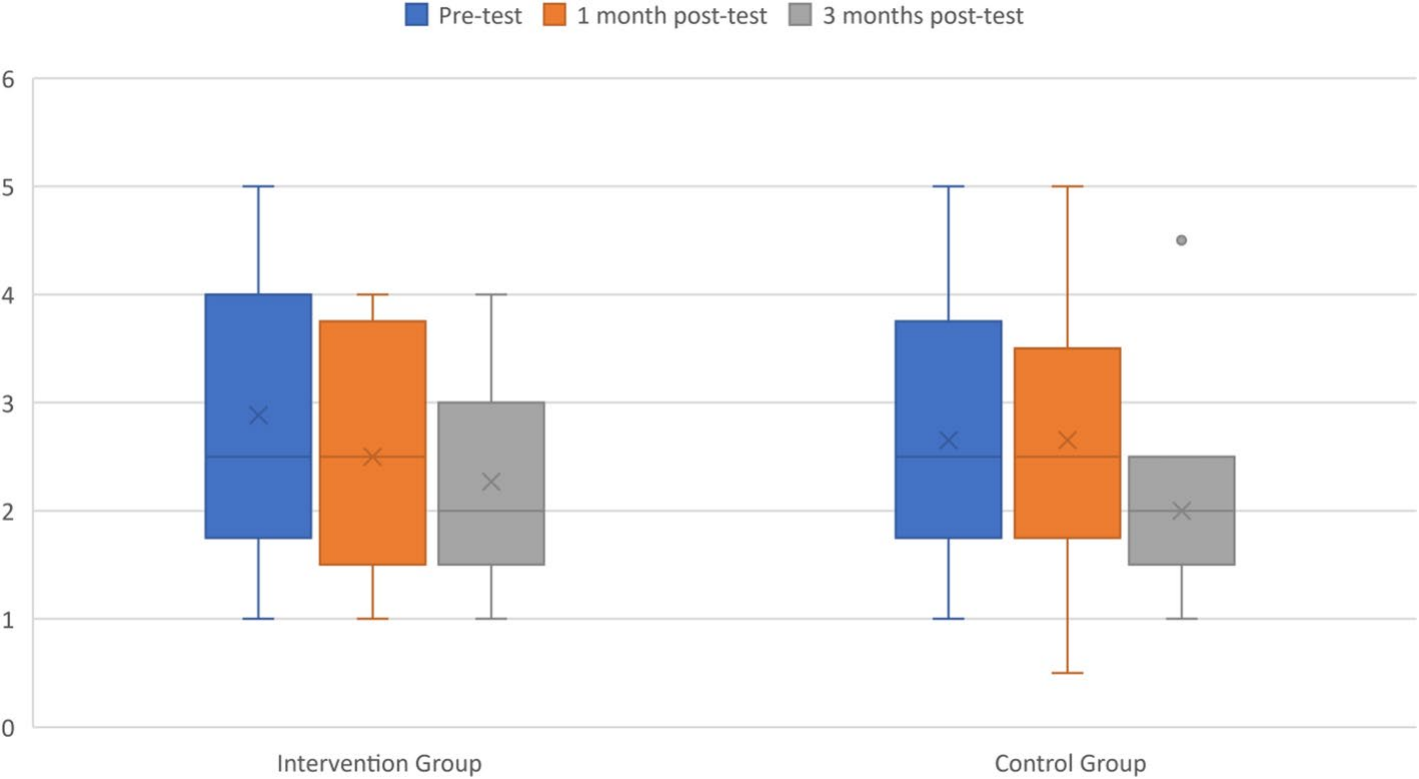
Evolution of ADL skills

**Fig. 5:**
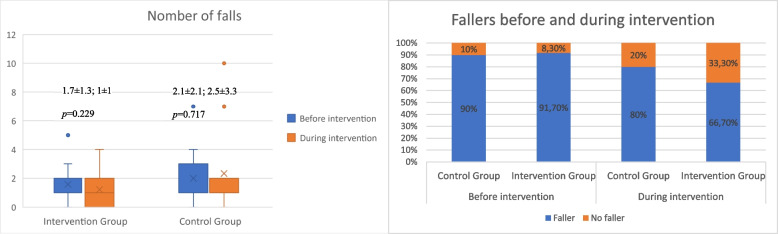
Evolution of falls and fallers

In terms of ADL abilities, both groups showed a decline, which was statistically significant only in the CG (F_(2)_ = 11.223, *p* < 0.001, ηp^2^ = 0.483, β-1 = 0.984). This substantial effect size reveals the extent of the decline for the CG. The Bonferroni test indicated that the CG's score on the Katz Index at the three-month post-test was significantly worse than at baseline (mean difference = -0.654, *p* = 0.005, d = 0.65). Figure 4 shows that while the IG's outcomes were dispersed, the CG's results were consistent and uniform, creating a significant difference in both post-tests for this group. Moreover, the IG reduced the average number of falls and the percentage of fallers during the 3-month intervention compared to the previous three months, whereas the CG increased the total number of falls. Nonetheless, no significant difference was found in the number of falls or fallers during the intervention, as depicted in Fig. 5. Lastly, no significant differences were found in the QoL-AD or in the SPPB, except in the gait speed sub score for the IG.

## Discussion

The objective of this randomised controlled study carried out on institutionalised older people with dementia, was to evaluate the effect of tango-therapy intervention in gait speed, functional mobility, balance, falls, ADL and QoL. First, it is crucial to emphasise the population's attributes. Despite acceptable physical mobility (all were able to walk 10 m unassisted), cognitive capacities were severely compromised, making communication extremely challenging. However, intervention attendance and well-being show that both interventions were well accepted and enjoyed. The results revealed that the IG improved the gait speed. Conversely, the CG exhibited a significant decline in functional mobility as measured by the TUG test, as well as in ADL skills.

Several studies show that gait speed is an indicator of various underlying pathophysiological processes [28], such as cognitive impairment [29], capable of predicting health status, risk of future functional decline, and future adverse events, including hospitalisation, level of care required, and mortality [28, 30]. Moreover, gait impairment is an independent risk factors for falls: each 0.1 m/s decrease in gait speed was associated with a 7% increased risk of falls [31]. During this study, the IG significantly improved their gait speed, thereby directly reducing the risk of falls. On the other hand, the CG, despite a slight improvement during the first month of the interventions, experienced a non-significant decline in their gait speed between the first and the last post-test. This non-significant deterioration in the CG, combined with the improvement seen in the IG, contributed to a significant intergroup difference favouring the IG by the end of the interventions.

In addition, the CG significantly worsened their functional mobility, measured through the TUG test. Like the gait speed test, the TUG is a valuable indicator of the state of physical health. Previous research has identified links between the TUG, risk of falling [32], and fear of falling [21]. These studies suggest that cognitive function is related to the sit-to-stand transition and turning subphases along with some gait metrics [33]. Future research on tango in this population should include an instrumented TUG test, to evaluate which are the precise parameters that can be improved through tango interventions.

Despite there being no statistically significant differences, the number of falls increased in the CG while it decreased in the IG. In addition, the number of fallers decreased in both groups, but more markedly in the IG. This is consistent with data from a metanalysis which concluded that those who engaged in dance-based activities had a statistically significant reduced risk of falling (37%) and number of falls (31%) compared with their peers who engaged in walking, seated exercise, general aerobics and other types of exercise [34]. This could be explained by an improvement in executive functions induced by tango interventions. Extensive research has shown that executive functions are strongly linked to falls [35], and as was already said, executive function is linked to specific TUG-subphases [21]. However, Hackney et al. [9] found that after 20 tango sessions, participants improved mobility, motor-cognitive function, and gait but not executive function. In the other hand, we have not found literature that supports the hypothesis regarding the effectiveness of tango as a tool for fall prevention in older individuals with dementia. To clarify these potential relationship, future research should include the analysis of the impact of tango on the fall rate and its relationship with executive functions.

Regarding the abilities to perform ADLs, both groups experienced a deterioration, but this decline was statistically significant only for the CG. Other studies have shown a stabilisation of self-care skills through dance interventions [36, 37]. For example, one study that compared the impact of a dance intervention with an activity of cognitive stimulation in older patients with dementia found that the dance group's ADLs were stabilised while the control group's abilities decreased significantly [38]. ADLs require multi-task performance, similarly to dance which engages the sensorial system, attention, memory, locomotion and social connection. This characteristic could explain the different outcomes between groups.

The mechanism by which the tango interventions improve or maintain these parameters is not clear, since factors affecting gait speed and functional mobility in older people with dementia are not entirely known. However, thanks to recent advances in the understanding of neuromotor function, we hypothesize that these results derive from three main reasons: the presence of music, the performance of dance and the particular characteristics of tango.

Postural control, general movement and gait are complex processes that associate the interaction of various parts of the central nervous system [39]. Previous studies have shown that to rehabilitate these processes, multimodal interventions, combining exercise with music produced more positive results than exercise alone [38, 40]. Neuroimaging studies have shown that music can activate a variety of brain regions beyond just the auditory cortex, including the prefrontal cortex, limbic system and motor cortex [41]. This suggests that music can have a profound impact on various cognitive and emotional processes, such as memory, emotion regulation and motor coordination. In addition, studies have shown that listening to music can activate the reward system in the brain, leading to the release of dopamine, producing pleasure and motivation [42].

Furthermore, the motor cortex, somatosensory cortex, basal ganglia, and cerebellum are all involved in various aspects of dance learning and performance [43] like controlling movement and providing sensory feedback. In addition, an activation of the mirror system when listening to music suggests that musical auditory perception can evoke motor representations, thus facilitating the relearning of lost motor schemata [44]. Overall, the involvement of these brain regions highlights the complex motor and sensory demands of dance performance and shows to what extent dance can be effective in the motor rehabilitation in neurological conditions such as Alzheimer disease and others dementias.

Tango dance has been shown to have potential benefits for gait rehabilitation in individuals with neurological disorders. Some studies have suggested that the specific characteristics of tango, such as its rhythmic structure, partner interaction, and focus on weight shifting and balance control, can help improve gait [45], postural control [46], and functional mobility [47]. Indeed, this style requires movement initiation and cessation, anticipation and transfers of body weight, multi-directional perturbations, dissociation, whole body coordination, varied speeds and rhythms. Furthermore, participants focus on foot placement and attention to partner, path of movement, and aesthetics [48, 49]. These characteristics could make tango an excellent tool for balance and gait rehabilitation, as well as for fall prevention in older adults with dementia [50].

Tango therapy has been largely used in Parkinson’s disease [51]. A study found that, compared with subjects who attended an exercise program, participants engaged in a 20-tango class program showed significant improvements in overall Unified Parkinson's Disease Rating Scale and had better balance and higher scores on the TUG [52]. Concerning the tango therapy effect in older people, a study showed an improvement of mobility, gait and motor-cognitive function after three months of an adapted tango program [9]. To the best of our knowledge, this is the first randomized controlled trial analysing the effects of the tango therapy on gait speed and functional mobility in older people with moderate and severe dementia. The results seem to indicate that this intervention produces significant benefits by maintaining and even improving the functional capacities.

Contrary to dance, physical exercise for older people is usually composed of monotonous and analytical movements, devoid of interest and motivation. In fact, previous studies have highlighted that physical exercises that included multi-sensory stimulation and multi-task activities have better results than physical programs with only static, resistance, and flexibility training [53]. Furthermore, a study comparing a dance program to conventional fitness found a significant increase in grey matter volume in the left precentral gyrus and in the parahippocampal region in the dance group [54]. Dance integrates psychosocial, cognitive and motor skills into one activity within an enjoyable social setting. Indeed, music stimulates the brain’s reward centres, while dance activates its sensory and motor circuits [55].

Regarding the effect on QoL, both interventions led to an improvement. However, these changes were not statistically significant. There was a notable improvement in both groups between the pre-test and the 1-month post-test, followed by a slight decline between the 1-month post-test and the 3-month post-test. This pattern might be explained by a 20-day interruption after the first post-test due to a Covid-19 outbreak, which affected the frequency and number of activities. The principal aim of non-pharmacological interventions is to enhance or maintain QoL. As in our previous study [13], and in accordance with other research, tango interventions seem to be an appropriate activity to achieve this objective, independently of benefits in mobility [56, 57]. In addition, interventions based on tango focus on the work of emotions, are rich in sensory stimuli, provide fun and feelings of connection, which may be key to improving QoL. The access to meaningful activities as well as maintenance of relationships and links with caregivers, have been identified as elements that exert a beneficial effect on the QoL of institutionalised older people [58]. To summarise, the combination of exercise, sensory enrichment, and social interaction during dance, might have a positive effect on QoL [59]. Further studies are needed to delve deeper into this potential relationship.

A major strength of this study was allowing for the participation of institutionalised older adults with dementia, an extremely vulnerable population, despite numerous ethical, legal and logistical obstacles. The methodological consistency of this study is another of its strengths, since it was possible to carry out a randomisation of the participants and compare the intervention with another non-pharmacological intervention, of proven efficacy in several conditions. Our study's findings underscore the potential benefits of incorporating dance interventions, such as tango, into care plans for older individuals with dementia. These interventions demonstrate improvements in gait speed and help prevent functional decline, highlighting their relevance in clinical practice. However, we also noted some limitations: the study sample size was small, the absence of data about pharmacotherapy, there was no follow-up to determine the long-term effects of the intervention, it was not possible to automatically measure spatiotemporal parameters of gait nor to blind the assessment. Future studies should try to increase the sample size, follow-up on participants for at least one year and minimise the risk of bias by using blinding techniques whenever possible. Additionally, there should be an assessment of specific cognitive functions, such as visuo-spatial abilities or constructional praxis and technical outcome measurements.

## Conclusion

Tango interventions proved effective in enhancing gait speed and in mitigating the decline in functional mobility and ADL skill capacities. These results seem to originate from three elements: the presence of music, the performance of dance and the particular characteristics of tango. Interventions in therapeutic tango are easily implementable and well received. Providing older people with dementia access to non-pharmacological interventions may be a successful strategy to prevent functional decline. This study aims to contribute to a change in perspective on aging and dementia, as even in advanced stages of dementia it is possible to live well and dance tango.

## Data Availability

The datasets used and/or analysed during the current study are available from the corresponding author on reasonable request.

## Abbreviations

ADLs: Activities of daily living
CG: Control group
IG: Intervention group
MMSE: Mini Mental State Examination
PLUs: Protected Life Units
QoL: Quality of life
QoL-AD: Quality of life in Alzheimer disease
SPPB: Short Physical Performance Battery
TUG: Timed Up and Go test
