# Correction: Tango‑therapy vs physical exercise in older people with dementia; a randomized controlled trial

**DOI:** 10.1186/s12877-026-07538-z

**Published:** 2026-05-08

**Authors:** Lucia Bracco, Arrate Pinto‑Carral, Linda Hillaert, France Mourey

**Affiliations:** 1 Inserm U1093‑Cognition, Action and Sensorimotor Plasticity, Faculty of Sport Sciences, University of Burgundy, Dijon, 21078 France; 2 SALBIS Research Group, Faculty of Health Sciences, Nursing and Physiotherapy Department, Universidad de Leon, Ponferrada, 24401 Spain; 3 Centre Hospitalier Geriatrique du Mont d’Or, Albigny Sur Saone, France

**Correction: BMC Geriatr 23**,** 693 (2023)**

**https://doi.org/10.1186/s12877-023-04342-x**

Following publication of the original article [1], the authors would like to correct the Ethics approval and consent to participate section.

The sentence currently reads:

**Ethics approval and consent to participate**

The study was conducted following the Declaration of Helsinki, was approved by the Research Ethics Committee of the University of Burgundy (CERUBFC-2022–09-29–031) and was registered at ClinicalTrials.gov (ID: NCT05744011). Each person likely to take part in this research was informed and a summary document on the main characteristics of the study was given during the inclusion visit. The written informed consent statement was given for immediate signature or after a reflection period. In case of doubt about the patient’s ability to give consent, caregivers and the healthcare team were asked to help measure the expression of the person’s autonomy and presumed wishes. In the case of persons under legal protection, the magistrate and the competent judge were informed, and authorization was requested.

The sentence should read:

**Ethics approval and consent to participate**

The study was conducted following the Declaration of Helsinki, was approved by the Research Ethics Committee of the University of Burgundy (CERUBFC-2022–09-29–031) and was registered at ClinicalTrials.gov (ID: NCT05744011). Each person likely to take part in this research was informed and a summary document on the main characteristics of the study was given during the inclusion visit. The written informed consent statement was given for immediate signature or after a reflection period. In case of doubt about the patient’s ability to give consent, caregivers and the healthcare team were asked to help measure the expression of the person’s autonomy and presumed wishes. In the case of persons under legal protection, the magistrate and the competent judge were informed, and authorization was requested.

The authors wish to clarify that although the study received approval from the University Research Ethics Committee (CER), under French regulatory classification the research should have been submitted to a Comité de Protection des Personnes (CPP). This administrative oversight does not affect participant consent, safety, or the scientific validity of the study.

The original article [1] has been corrected.
